# Stress Pathways in Chronic Kidney Disease: Linking Cortisol, Oxidative Stress, and Inflammation

**DOI:** 10.3390/antiox14101259

**Published:** 2025-10-20

**Authors:** Maria Motrenikova, Krasimir Boyanov, Neli Bojinova, Anelia Bivolarska

**Affiliations:** 1 Department of Medical Biochemistry, Faculty of Pharmacy, Medical University of Plovdiv, Vasil Aprilov Str. 15A, 4002 Plovdiv, Bulgaria; krasimir.boyanov@mu-plovdiv.bg; 2 Dietary Nutrition Instructor, Medical College, Medical University of Plovdiv, Bratya Bukston Str. 120, 4004 Plovdiv, Bulgaria; neli.bozhinova@mu-plovdiv.bg

**Keywords:** chronic kidney disease, cortisol, oxidative stress, inflammation, PNEI axis, chronic stress, HPA dysregulation, antioxidant defense

## Abstract

This review aims to synthesize current evidence on the role of chronic stress and hypothalamic–pituitary–adrenal (HPA) axis dysregulation in the pathogenesis of chronic kidney disease (CKD). The focus is on the interplay between cortisol, oxidative stress, inflammation, and metabolic risk factors within the psycho-neuro-endocrine-immune (PNEI) system. CKD is a multifactorial disease characterized by oxidative stress, chronic low-grade inflammation, and neuroendocrine imbalance. These processes interact to accelerate renal injury and systemic complications. Pro-inflammatory mediators such as tumor necrosis factor-alpha (TNF-α), interleukin-1 beta (IL-1β), and interleukin-6 (IL-6), together with oxidative stress markers including malondialdehyde (MDA), advanced oxidation protein products (AOPPs), and 8-hydroxy-2′-deoxyguanosine (8-OHdG), are strongly associated with disease progression. Altered cortisol dynamics—assessed in serum, saliva, and hair—further reflect chronic HPA activation and contribute to immune dysfunction, metabolic disturbances, and cardiovascular risk. By integrating experimental and clinical findings, this review highlights how stress-induced dysregulation of the PNEI system amplifies CKD progression. Understanding these interconnected mechanisms underscores the potential of combining oxidative, inflammatory, and neuroendocrine biomarkers for improved risk stratification and targeted therapeutic interventions.

## 1. Introduction

CKD is a progressive and multifactorial disorder that affects more than 10% of the global adult population, representing a major public health and socioeconomic challenge due to its high morbidity, mortality, and treatment costs [1,2]. Beyond its traditional risk factors, CKD is increasingly recognized as a disease of premature biological aging, driven by persistent activation of damaging molecular pathways, including oxidative stress, low-grade systemic inflammation, and hormonal dysregulation [3,4].

Chronic low-grade inflammation is a central feature of CKD and is associated with accelerated vascular damage, atherosclerosis, and mortality. Large population-based studies have shown that systemic inflammatory markers such as the systemic immune-inflammation index (SII) and neutrophil-to-lymphocyte ratio predict CKD prevalence and outcomes [5]. Similarly, oxidative stress plays a pivotal role by promoting endothelial dysfunction, tubular injury, and fibrosis. Novel biomarkers such as advanced oxidation protein products (AOPPs) and thiobarbituric acid reactive substances (TBARSs) have been proposed as predictors of CKD progression [6,7].

Dysregulation of HPA axis contributes to the chronic stress response in CKD. Excess cortisol exposure has been associated with metabolic disturbances, immune dysregulation, and increased mortality in CKD patients [8]. Moreover, hair cortisol has recently been validated as a biomarker of long-term HPA axis activity and shown to correlate with kidney and cardiometabolic parameters in CKD [9]. Understanding the interplay between chronic stress, HPA axis activation, oxidative stress, and inflammation is essential for identifying novel biomarkers and designing targeted interventions to slow CKD progression.

**Figure 1:**
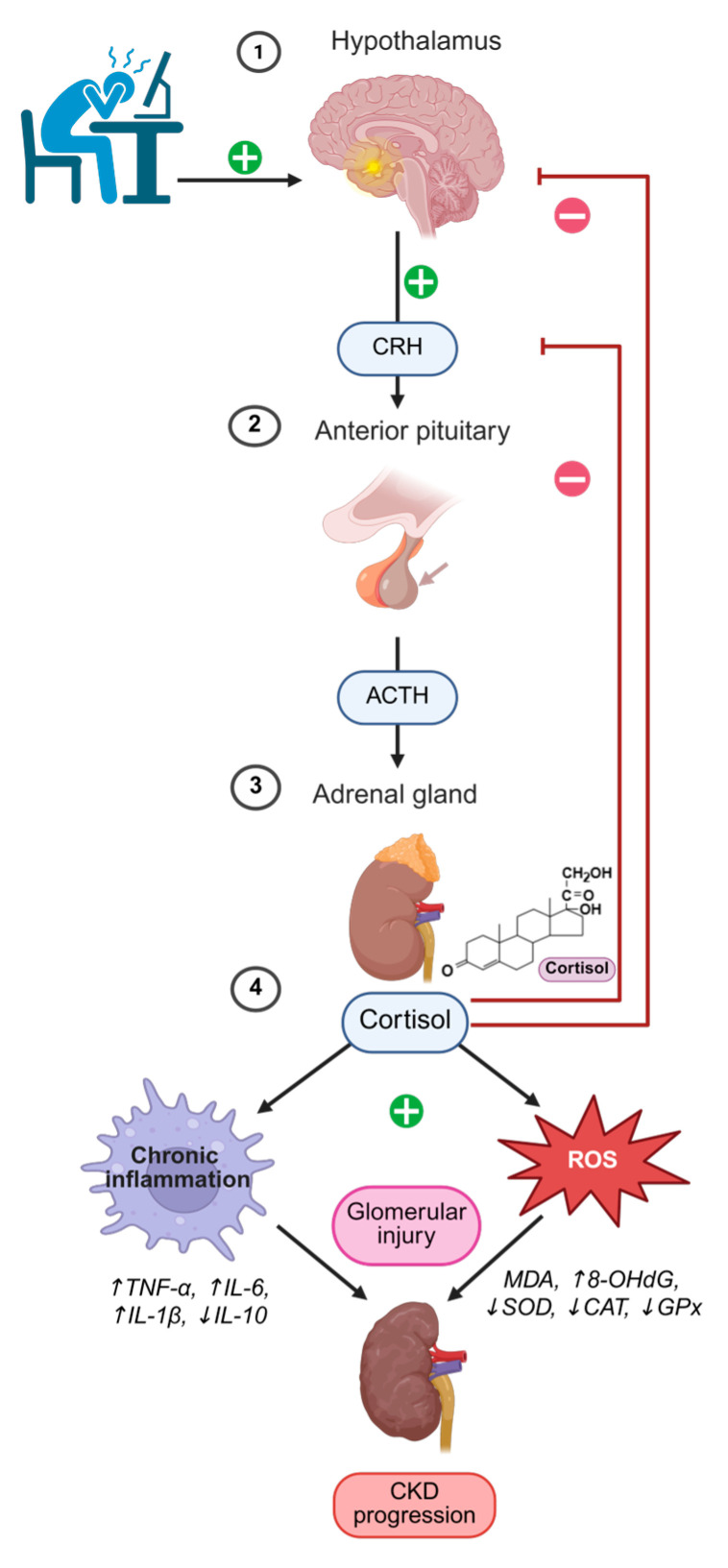
Dysregulation of the HPA/PNEI axis and its contribution to chronic kidney disease progression.

This review aims to synthesize current evidence on the interactions between chronic stress, HPA axis dysregulation, oxidative stress, and inflammation in CKD, highlighting their mechanistic links and potential clinical implications (Figure 1).

The literature search for this review was conducted in PubMed, Scopus, and Web of Science databases for articles published between 2010 and 2025 using keywords including “chronic kidney disease”, “cortisol”, “oxidative stress”, “inflammation”, and “HPA axis”. Preference was given to original articles, systematic reviews, and meta-analyses published within the last five years. Older but highly cited landmark studies were also included when essential for explaining core mechanisms.

The figure illustrates how chronic stress activates the psycho-neuro-endocrine-immune (PNEI) system via the hypothalamic–pituitary–adrenal (HPA) axis. Stress stimulates the hypothalamus to release corticotropin-releasing hormone (CRH), which activates the anterior pituitary to secrete adrenocorticotropic hormone (ACTH). ACTH in turn stimulates the adrenal glands to produce cortisol [10]. Excess cortisol modulates immune and metabolic pathways, leading to chronic inflammation (↑ tumor necrosis factor-alpha (TNF-α), ↑ interleukin 6 (IL-6), ↑ interleukin 1 beta (IL-1β), ↓ interleukin 10 (IL-10)) and oxidative stress (↑ reactive oxygen species (ROS), ↑ malondialdehyde (MDA), ↑ 8-hydroxy-2′-deoxyguanosine (8-OHdG), ↓ superoxide dismutase (SOD), ↓ catalase (CAT), ↓ glutathione peroxidase (GPx)). These processes interact in a vicious cycle, amplifying each other, and ultimately contribute to glomerular injury and the progression of chronic kidney disease (CKD).

Created with BioRender.com. (https://www.biorender.com, accessed on 28 September 2025)

## 2. Pathophysiological Links Between CKD and Stress Response

### 2.1. Oxidative Stress in CKD

Oxidative stress (OS) is a fundamental pathogenic mechanism in CKD, arising when ROS generation exceeds the capacity of endogenous antioxidant systems. Persistent uremic conditions amplify ROS production through mitochondrial respiratory chain dysfunction and the activation of pro-oxidant enzymes, including NADPH oxidases (NOX4 and the human-specific NOX5), xanthine oxidase, myeloperoxidase, and uncoupled endothelial nitric oxide synthase (eNOS) [11]. These processes drive cellular injury and inflammation, while simultaneously depleting protective enzymatic antioxidants such as SOD, CAT, and GPx, as well as non-enzymatic systems including GSH and vitamins [12]. As renal function declines, this redox imbalance becomes progressively more pronounced, contributing to tubular atrophy, glomerulosclerosis, and the acceleration of cardiovascular complications [13]. Moreover, oxidative stress in CKD extends beyond renal damage and represents a major driver of cardiovascular complications. The excessive generation of ROS promotes endothelial dysfunction, vascular inflammation, and myocardial remodeling, thereby linking renal and cardiovascular pathology within a shared oxidative–inflammatory axis [14] (Table 1).

Numerous biomarkers reflect this systemic oxidative burden and are increasingly applied in clinical studies. Among them, lipid peroxidation markers such as MDA and F2-isoprostanes are consistently elevated across all CKD stages and show inverse correlations with estimated glomerular filtration rate (eGFR) [15]. Protein oxidation markers, notably advanced oxidation protein products (AOPPs) and protein carbonyl derivatives, accumulate in plasma and correlate with both inflammatory status and renal fibrosis [16]. Oxidative DNA damage, measured by 8-OHdG, has emerged as a sensitive indicator of cumulative ROS exposure and predicts faster progression and higher cardiovascular risk among CKD patients [17]. Beyond individual molecules, composite indices such as the oxidative balance score (OBS), which integrates dietary intake and circulating pro- and antioxidant biomarkers, have shown inverse associations with CKD incidence and progression in population-based cohorts [18]. These findings underscore the clinical potential of standardized redox biomarker panels to improve risk stratification and disease monitoring.

**Figure 2:**
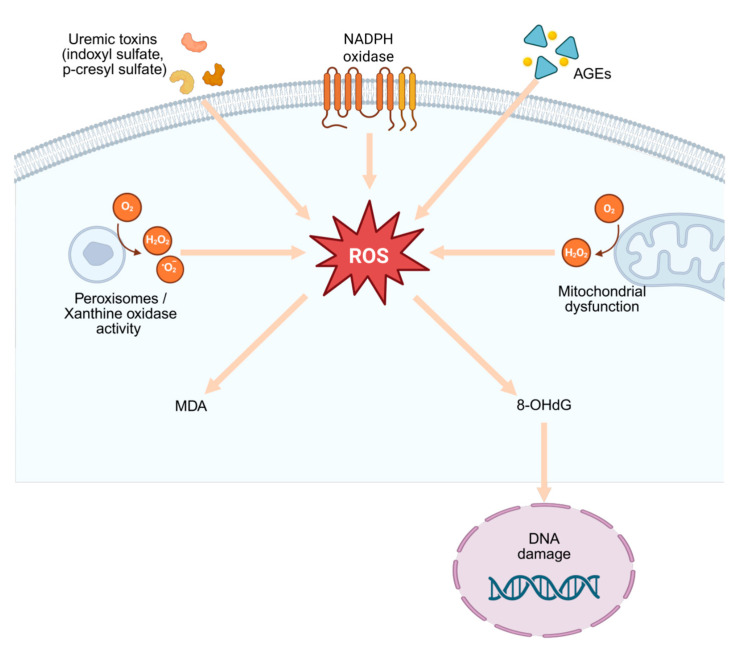
Sources and consequences of oxidative stress in chronic kidney disease.

Mechanistically, oxidative stress does not merely accompany CKD but actively perpetuates its progression. Mitochondrial dysfunction, characterized by impaired biogenesis, disrupted dynamics, and defective mitophagy, drives sustained ROS generation and activates profibrotic and inflammatory signaling cascades, including NF-κB and the NLRP3 inflammasome [19]. Recent evidence emphasizes that mitochondrial-derived reactive oxygen species (mtROS) are a dominant source of oxidative damage in CKD. Impaired mitochondrial respiration, altered dynamics, and defective mitophagy promote chronic inflammation, fibrosis, and tubular injury [20,21]. In parallel, hyperphosphataemia stimulates NOX activity and endothelial injury, creating a feed-forward cycle of vascular damage and redox imbalance [22]. A growing body of evidence highlights the distinct contributions of NADPH oxidase isoforms: while NOX4 appears to play a dual role, promoting both injury and protective redox signaling, endothelial NOX5—absent in rodents but present in humans—exacerbates diabetic kidney injury and negates the beneficial effects of NOX4 deletion [23]. Such findings illustrate the complexity of ROS-related pathways in CKD and the need for precise mechanistic dissection to guide targeted interventions (Figure 2).

Therapeutic efforts to counteract oxidative stress are evolving from non-specific antioxidant supplementation toward mechanism-based strategies. Experimental studies have demonstrated that selective NOX4 inhibition preserves mitochondrial function and reduces ischemia–reperfusion kidney injury [24], while nutritional interventions using polyphenol- and flavonoid-rich diets have shown promising antioxidant effects in both preclinical and early clinical CKD studies [25]. Recent reviews, however, emphasize that the benefits of antioxidant therapy depend on careful targeting, timing, and dosing, cautioning against indiscriminate high-dose supplementation [26]. Activation of the nuclear factor erythroid 2–related factor 2 (Nrf2) pathway—a master regulator of cellular redox homeostasis—has attracted interest as Nrf2 activity is frequently downregulated in CKD, although responses appear context-dependent across disease models [27]. Finally, sodium-glucose cotransporter-2 (SGLT2) inhibitors, now widely used in diabetic and non-diabetic CKD, consistently reduce circulating oxidative and inflammatory markers alongside their established renoprotective effects, suggesting that redox modulation contributes to their clinical efficacy [28]. Beyond renal injury, oxidative stress represents a critical mechanistic link between CKD and cardiovascular diseases (CVDs). Persistent redox imbalance contributes to endothelial dysfunction, vascular calcification, and accelerated atherosclerosis—processes that substantially increase morbidity and mortality in CKD patients. Clinical and experimental studies consistently demonstrate that elevated oxidative stress markers such as 8-OHdG and AOPPs are associated with impaired vascular compliance and adverse cardiovascular outcomes [14]. These findings highlight the bidirectional relationship between oxidative stress, renal impairment, and cardiovascular pathology.

Together, these findings position oxidative stress not simply as a by product but as a mechanistic contributor to CKD. Reliable assessment using validated biomarkers such as MDA, AOPPs, and 8-OHdG, complemented by composite indices like the OBS, combined with strategies that target upstream ROS sources and restore endogenous antioxidant defenses, may open new avenues to slow disease progression and improve clinical outcomes.

**Table 1:** Oxidative stress in CKD: sources, biomarkers, mechanisms, and therapeutic strategies.

Aspect	Mechanism/Effect	References
ROS sources in CKD	Mitochondrial dysfunction, NOX4/NOX5, xanthine oxidase, myeloperoxidase, uncoupled eNOS; amplified under uremic conditions.	[11]
Antioxidant defense impairment	Depletion of enzymatic antioxidants (SOD, CAT, GPx) and non-enzymatic systems (GSH, vitamins).	[12]
Lipid peroxidation biomarkers	MDA, F2-isoprostanes ↑ with CKD stage, inversely correlate with eGFR.	[15]
Protein oxidation biomarkers	AOPPs and protein carbonyls ↑, associated with inflammation and renal fibrosis.	[16]
DNA oxidation biomarker	8-OHdG reflects cumulative ROS burden, predicts progression and cardiovascular risk.	[17]
Composite indices	OBS: integrates diet and biomarkers, inversely associated with CKD incidence/progression.	[18]
Mitochondrial dysfunction	Impaired biogenesis, mitophagy, and dynamics drive ROS overproduction; activate NF-κB and NLRP3 inflammasome.	[19,20,21]
Phosphate-driven stress	Hyperphosphataemia activates NOX, causes endothelial injury, amplifies vascular damage.	[22]
Role of NOX isoforms	NOX4 has dual injury/protective role; endothelial NOX5 (human-specific) worsens diabetic kidney injury.	[23]
Mechanism-based therapies	Selective NOX4 inhibition preserves mitochondria and reduces injury.	[24]
Nutritional antioxidants	Polyphenol/flavonoid-rich diets show protective effects in CKD.	[25]
Targeting redox regulation	Nrf2 pathway activation restores cellular redox balance; context-dependent effects.	[27]
Clinical pharmacotherapy	SGLT2 inhibitors lower oxidative/inflammatory markers and improve renal outcomes.	[28]

Legend: This table summarizes major sources of oxidative stress in CKD, associated biomarkers, pathophysiological mechanisms, and emerging therapeutic approaches. The arrow symbol (↑) indicates an increase in biomarker levels with CKD stage.

Reactive oxygen species (ROS) are generated from multiple cellular sources, including mitochondrial dysfunction and peroxisomal/xanthine oxidase activity. Excessive ROS production leads to lipid peroxidation, represented by malondialdehyde (MDA), and oxidative DNA damage, marked by 8-hydroxy-2′-deoxyguanosine (8-OHdG). These processes contribute to DNA injury and play a central role in the pathogenesis and progression of chronic kidney disease (CKD).

Created with BioRender.com. (https://www.biorender.com, accessed on 28 September 2025)

### 2.2. Inflammation and CKD

CKD is characterized by a state of chronic low-grade inflammation, defined as a persistent, systemic immune activation with mildly elevated levels of pro-inflammatory mediators that fail to resolve over time [29]. This subclinical inflammatory state contributes to both the onset and progression of CKD by sustaining tissue injury, promoting fibrosis, and accelerating cardiovascular comorbidities (Table 2).

**Figure 3:**
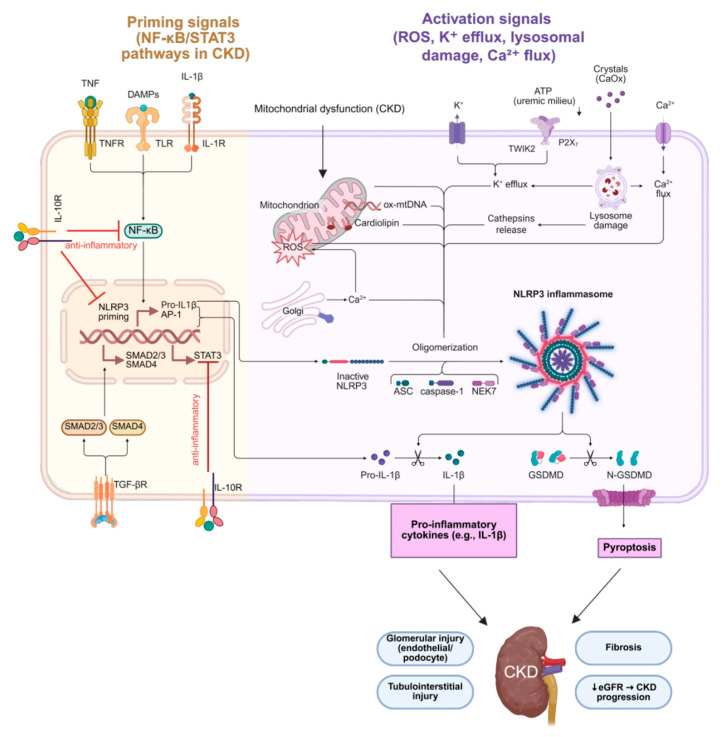
Priming and activation of the NLRP3 inflammasome in chronic kidney disease.

A major driver of inflammation in CKD is the activation of the NLRP3 inflammasome, particularly in renal myeloid cells, leading to increased production of IL-1β (Figure 3). IL-1β amplifies local and systemic inflammation and accelerates atherosclerosis, thereby indirectly aggravating nephropathy [30]. The downstream activation of JAK/STAT and NF-κB signaling further sustains the inflammatory cascade by upregulating adhesion molecules, chemokines, and profibrotic factors such as TGF-β [31]. In parallel, aryl hydrocarbon receptor (AhR) signaling, increasingly recognized in renal pathology, has been shown to mediate both oxidative stress and inflammation, and its overactivation promotes glomerular injury and tubular cell apoptosis in CKD [32].

Among the circulating cytokines, IL-6 plays a central role as a pleiotropic pro-inflammatory mediator linking CKD, diabetes, and cardiovascular disease [33]. Experimental and clinical data demonstrate that sustained IL-6 expression correlates with glomerulosclerosis, endothelial dysfunction, and declining eGFR. In a recent study of patients with CKD stages 3–5, elevated IL-6 and C-reactive protein (CRP) levels were significantly associated with lower eGFR [34]. Blocking IL-6 signaling is currently explored as a therapeutic strategy to attenuate CKD progression [35].

Tumor necrosis factor-alpha (TNF-α) is another key cytokine driving renal inflammation. It is secreted not only by immune cells but also by renal tubular epithelial and mesangial cells, as well as vascular endothelial cells, where it induces IL-1β and IL-6 expression through NF-κB and p38 MAPK pathways [36]. In CKD, persistently elevated TNF-α contributes to endothelial dysfunction, microvascular damage, and proteinuria [37]. Notably, a novel mechanism has been described where granulosa cell-derived TNF-α induces inflammation and apoptosis of renal tubular cells, mediating PCOS-related kidney injury via NF-κB signaling [38]. Moreover, lin28a overexpression in nephron compartments has been shown to trigger a strong inflammatory response and aggravate renal damage [39].

Inflammatory signaling in CKD is further amplified by gut microbiota dysbiosis, which promotes the translocation of endotoxins and microbial metabolites that activate toll-like receptors (TLRs), stimulate oxidative stress, and drive chronic systemic inflammation [40]. Chk2, a DNA damage response kinase, was recently implicated as a regulator of renal aging and fibrosis, linking oxidative stress, inflammation, and epithelial–mesenchymal transition (EMT) [41]. Likewise, IGF2BP1 inhibition has been reported to reduce renal injury by attenuating inflammation through decreased E2F1/MIF pathway activity and m6A RNA modifications [42].

Counterbalancing these pro-inflammatory pathways, interleukin-10 (IL-10) acts as a major anti-inflammatory cytokine that suppresses TNF-α, IL-1β, and IL-6 production while promoting tissue repair [43]. Reduced IL-10 expression is commonly observed in CKD and is associated with uncontrolled inflammation and progressive renal fibrosis. The IL-10/TGF-β axis thus represents an important compensatory mechanism attempting to limit inflammatory damage during CKD progression.

Overall, CKD represents a state of chronic low-grade inflammation fueled by multiple interconnected mechanisms, including classical cytokine networks, dysregulated immune sensing pathways, metabolic and oxidative stress, and emerging regulatory molecules. Understanding these inflammatory drivers may open opportunities for novel targeted interventions to slow CKD progression.

**Table 2:** Inflammation in CKD: key pathways, mediators, and clinical implications.

Aspect	Mechanism/Effect	References
Chronic low-grade inflammation	Persistent systemic immune activation with elevated cytokines, driving fibrosis and cardiovascular comorbidities.	[29]
NLRP3 inflammasome	Activated in renal myeloid cells → ↑ IL-1β → amplifies inflammation and accelerates atherosclerosis.	[30]
Downstream signaling	JAK/STAT and NF-κB activation → adhesion molecules, chemokines, TGF-β (profibrotic).	[31]
AhR signaling	Mediates oxidative stress and inflammation; overactivation promotes glomerular injury and tubular apoptosis.	[32]
IL-6	Central cytokine linking CKD, diabetes, and CVD; ↑ IL-6 and CRP correlate with low eGFR; therapeutic target.	[33,34,35]
TNF-α	Produced by immune, renal, and endothelial cells; drives IL-1β/IL-6 via NF-κB and MAPK; ↑ endothelial dysfunction and proteinuria.	[36,37]
Novel TNF-α mechanisms	Granulosa cell–derived TNF-α induces tubular apoptosis (PCOS-related); lin28a overexpression aggravates renal inflammation.	[38,39]
Gut microbiota dysbiosis	Endotoxin and metabolite translocation → TLR activation, oxidative stress, systemic inflammation.	[40]
IL-10 (anti-inflammatory)	Suppresses TNF-α, IL-1β, IL-6; reduced IL-10 in CKD linked with uncontrolled inflammation and fibrosis.	[43]

Legend: This table summarizes the main inflammatory mechanisms in CKD, highlighting classical cytokines, immune signaling pathways, gut microbiota effects, and emerging molecular regulators. Arrows (↑) indicate causative or sequential relationships between processes, representing increased IL-1β and IL-6 production and enhanced endothelial dysfunction.

Priming signals in chronic kidney disease (CKD), including tumor necrosis factor-α (TNF-α), interleukin-1β (IL-1β), and damage-associated molecular patterns (DAMPs), activate the nuclear factor kappa B (NF-κB) and signal transducer and activator of transcription 3 (STAT3) pathways, leading to transcriptional upregulation of nucleotide-binding domain leucine-rich repeat-containing receptor family pyrin domain containing 3 (NLRP3) and pro-IL-1β. Transforming growth factor-β (TGF-β)/SMAD signaling and interleukin-10 (IL-10) exert counter-regulatory anti-inflammatory effects. Activation signals such as mitochondrial reactive oxygen species (ROS), potassium efflux (K^+^), calcium flux (Ca^2+^), lysosomal destabilization, and uremic crystals promote NLRP3 inflammasome oligomerization with apoptosis-associated speck-like protein containing a CARD (ASC), caspase-1, and NIMA-related kinase 7 (NEK7). This results in maturation of IL-1β, cleavage of gasdermin D (GSDMD), and pyroptosis, releasing pro-inflammatory mediators. These processes contribute to glomerular (endothelial and podocyte) and tubulointerstitial injury, fibrosis, and progressive decline in estimated glomerular filtration rate (eGFR), ultimately driving CKD progression. ↓ eGFR indicates decreased estimated glomerular filtration rate.

Figure created with BioRender.com. (https://www.biorender.com, accessed on 28 September 2025)

### 2.3. HPA/PNEI Axis Dysregulation and CKD

CKD is increasingly recognized as not only a renal disorder but also a condition tightly interwoven with systemic neuroendocrine and immune dysfunction. The HPA axis forms a central component of the broader PNEI network, which coordinates the organism’s response to stressors. Persistent activation of the HPA axis in the setting of chronic psychosocial or physiological stress drives sustained secretion of glucocorticoids—primarily cortisol—which exerts pleiotropic effects on metabolism, immunity, and cardiovascular regulation [9]. Dysregulation of this axis is a hallmark of chronic stress, and accumulating evidence indicates that such dysregulation contributes to the onset and progression of CKD [44].

Under normal circumstances, cortisol secretion follows a circadian pattern, peaking shortly after awakening and gradually declining toward the evening. In CKD, this rhythm is frequently blunted or phase-shifted, with a diminished morning peak and relatively elevated evening cortisol levels [45]. Such alterations may reflect both impaired negative feedback within the HPA axis and altered peripheral cortisol metabolism due to reduced renal clearance and enhanced 11β-hydroxysteroid dehydrogenase type 1 (11β-HSD1) activity [46]. Elevated cortisol exposure promotes visceral adiposity, insulin resistance, and hypertension—established metabolic risk factors for CKD—and simultaneously enhances oxidative stress and pro-inflammatory cytokine release, thereby perpetuating kidney injury [47].

Mounting evidence supports the existence of bidirectional crosstalk between HPA axis activity and immune responses in CKD. Glucocorticoids normally exert anti-inflammatory actions, suppressing the transcription of pro-inflammatory cytokines such as TNF-α, IL-1β, and IL-6. However, chronic cortisol excess induces a state of glucocorticoid resistance in immune cells, paradoxically resulting in heightened inflammatory responses [48]. Dysregulated cortisol signaling has been associated with increased circulating levels of IL-6, TNF-α, and C-reactive protein in patients with CKD, further linking HPA axis dysfunction to the chronic low-grade inflammatory milieu characteristic of the disease [46]. The combined disruption of neuroendocrine and immune homeostasis thus represents a crucial mechanistic pathway bridging chronic stress and CKD progression.

Prolonged dysregulation of the HPA axis in CKD does not act in isolation but interacts closely with the sympathetic nervous system (SNS) and the immune system, forming the core of the PNEI network. Chronic activation of the SNS augments renin–angiotensin–aldosterone system (RAAS) signaling, which increases blood pressure and contributes to glomerular hyperfiltration and fibrosis [9,49]. Elevated sympathetic tone also enhances oxidative stress through activation of NADPH oxidases, thereby linking neuroendocrine imbalance with renal oxidative damage [50].

The maladaptive PNEI response further amplifies inflammation in CKD. Cortisol excess, combined with heightened catecholamine signaling, alters T-cell differentiation and macrophage polarization, favoring a pro-inflammatory milieu dominated by Th1/Th17 cells and M1 macrophages [51]. This imbalance promotes secretion of IL-6, TNF-α, and other pro-inflammatory cytokines, which have been shown to upregulate 11β-HSD1 in various tissues, including renal cells, thereby potentially sustaining local cortisol regeneration and creating a self-reinforcing loop of inflammation and stress hormone activation [46,52].

Emerging data highlight that HPA/PNEI dysregulation also impacts metabolic and cardiovascular comorbidities that accelerate CKD progression. Altered cortisol dynamics are associated with increased visceral adiposity, insulin resistance, and dyslipidemia, which contribute to endothelial dysfunction and microvascular injury [53,54,55]. Furthermore, aberrant glucocorticoid and catecholamine signaling have been linked to sleep disturbances and depressive symptoms in CKD patients, further destabilizing HPA-axis regulation and fostering chronic low-grade inflammation [56,57].

Together, these findings underscore that CKD progression is driven not only by classical renal pathophysiology but also by systemic dysregulation of stress-response pathways. Interventions such as aerobic exercise, which attenuate sympathetic overactivation and improve vascular function, have shown beneficial effects in CKD patients and may contribute to slowing disease progression [58].

Beyond its mechanistic role, dysregulation of the HPA/PNEI axis in CKD offers opportunities for therapeutic intervention. Pharmacological targeting of glucocorticoid pathways—particularly through mineralocorticoid receptor antagonists—has shown promise in mitigating cortisol/aldosterone-driven inflammation and fibrosis, thereby improving renal outcomes without fully suppressing physiological stress responses [59]. Likewise, inhibition of pro-inflammatory cytokines, particularly IL-6 (for example, via monoclonal antibody therapy in CKD patients), has been proposed as a strategy to attenuate the vicious cycle linking stress hormones and systemic inflammation [60,61]. In addition to pharmacological therapies, lifestyle interventions such as structured aerobic and combined exercise programs have demonstrated beneficial effects on inflammation, vascular function, and quality of life in CKD, with possible benefits on stress hormone regulation [62,63] (Table 3).

Importantly, the PNEI perspective underscores the need for an integrative therapeutic framework: CKD management should not be limited to renoprotective and metabolic strategies, but also actively address neuroendocrine–immune imbalance. Incorporating stress physiology into clinical nephrology, for example, through monitoring cortisol dynamics, could enable earlier detection of maladaptive HPA activity, guide personalized interventions, and ultimately contribute to slowing CKD progression and improving patient outcomes [9,44].

### 2.4. Metabolic Risk Factors and CKD

Metabolic risk factors play a central role in the onset and progression of CKD. Among these, hypertension, diabetes mellitus, obesity, and dyslipidemia are the most significant contributors, frequently coexisting and interacting synergistically to accelerate renal decline.

#### 2.4.1. Hypertension in CKD

Hypertension is both a cause and consequence of CKD, forming a self-perpetuating cycle of renal and vascular injury. Elevated systemic blood pressure promotes glomerular hypertension and hyperfiltration, leading to nephron loss and fibrosis [64]. Conversely, CKD impairs sodium handling and activates the RAAS, further worsening blood pressure control [65]. Novel insights highlight the role of RAAS-independent mechanisms, such as sympathetic overactivity and endothelial dysfunction, in driving hypertension-related renal injury [54,66].

#### 2.4.2. Diabetes in CKD

Diabetes mellitus is the leading cause of CKD worldwide, with up to 40% of patients with type 2 diabetes developing diabetic kidney disease (DKD) [67]. Hyperglycemia promotes the formation of advanced glycation end-products (AGEs), oxidative stress, and microvascular inflammation, which drive glomerulosclerosis and tubulointerstitial fibrosis [68]. Both type 1 and type 2 diabetes are implicated, although the prevalence of DKD is rising more steeply in type 2 diabetes due to increasing global obesity and aging [69]. Recent studies emphasize that, in addition to glycemic control, therapies targeting metabolic and inflammatory pathways, such as SGLT2i and glucagon-like peptide-1 receptor agonists (GLP-1RA), provide significant renoprotection. Evidence from large randomized clinical trials, including the FLOW trial, has demonstrated that GLP-1RA therapy reduces kidney outcomes beyond glucose lowering, while SGLT2i consistently improve both renal and cardiovascular endpoints [70,71,72].

#### 2.4.3. Obesity in CKD

Obesity is an independent metabolic risk factor for CKD development and progression. Mechanistically, it contributes to renal injury through hemodynamic changes, ectopic fat deposition in the kidney, chronic inflammation, and activation of RAAS [73]. Obesity-related glomerulopathy is characterized by glomerulomegaly, proteinuria, and progressive loss of renal function [74]. Moreover, obesity is closely associated with other CKD risk factors, such as diabetes, hypertension, and dyslipidemia, amplifying the overall risk burden [75]. Lifestyle interventions, including caloric restriction and exercise, as well as pharmacological treatments targeting weight loss, have demonstrated beneficial effects on renal outcomes in obese CKD patients [76,77,78].

#### 2.4.4. Dyslipidemia and Metabolic Syndrome in CKD

Dyslipidemia, characterized by elevated triglycerides, reduced HDL cholesterol with impaired anti-atherogenic function, and qualitative LDL changes such as an increase in small dense LDL particles, is increasingly recognized as a contributor to CKD progression and its associated cardiovascular burden [79,80]. Lipid accumulation in the kidney promotes oxidative stress, podocyte injury, and inflammation, thereby contributing to glomerulosclerosis and renal lipotoxicity. Recent evidence indicates that mitochondrial dysfunction and excessive mtROS production represent a key link between lipid abnormalities, oxidative stress, and chronic inflammation in CKD. Altered mitochondrial dynamics and impaired fatty acid oxidation contribute to dysregulated lipid handling, while accumulated lipotoxic intermediates further amplify oxidative injury and endothelial dysfunction [21,22].

Dysregulation of lipid metabolic regulators such as peroxisome proliferator-activated receptors (PPARs) and sterol regulatory element-binding protein 1 (SREBP-1) further aggravates lipid deposition and fibrotic remodeling. The coexistence of dyslipidemia with central obesity, insulin resistance, and hypertension defines the metabolic syndrome, which strongly predisposes to CKD [81,82]. Importantly, lipid-lowering therapies such as statins reduce cardiovascular risk in CKD but have demonstrated variable effects on renal outcomes. Recent genetic analyses of lipid-lowering drug target genes highlight the potential for novel therapeutic approaches specifically addressing renal lipotoxicity [83,84]. Emerging mechanistic evidence highlights that mitochondrial dysfunction and excessive generation of reactive oxygen species (ROS) are key factors linking dyslipidemia to renal injury and fibrosis [85]. Lipid accumulation within renal tubular and glomerular cells enhances oxidative stress and disrupts fatty acid β-oxidation, thereby amplifying redox imbalance and inflammation. These processes contribute not only to renal lipotoxicity but also to endothelial dysfunction, vascular calcification, and atherosclerotic remodeling, establishing a mechanistic bridge between chronic kidney disease and cardiovascular pathology [79]. Mitochondria-targeted antioxidants and agents that improve lipid metabolism and redox balance within the kidney are currently being explored as potential therapeutic strategies to interrupt this harmful interplay [79,86].

Collectively, these metabolic risk factors rarely act in isolation; rather, they converge through shared mechanisms such as oxidative stress, chronic inflammation, endothelial dysfunction, and lipotoxicity [48,49,79]. Their frequent coexistence creates a synergistic environment that accelerates CKD onset and progression, highlighting the importance of integrated management strategies.

### 2.5. CKD and Accelerated Aging

Physiological aging is accompanied by a gradual decline in renal function, particularly a reduction in glomerular filtration rate (GFR) and renal plasma flow [82]. In patients with CKD, however, these processes occur prematurely and with greater severity, establishing CKD as a clinical model of accelerated biological aging. Several molecular pathways implicated in aging and premature vascular aging in CKD include Nrf2, AMP-activated protein kinase (AMPK), sirtuin 1 (SIRT1), and the anti-aging protein klotho, all of which represent potential therapeutic targets [87]. The premature aging phenotype in CKD is closely linked to vascular changes, including arteriosclerosis, endothelial dysfunction, and vascular calcification, which substantially contribute to the elevated cardiovascular morbidity and mortality observed in this population [88]. On a molecular level, uremic arteries demonstrate a senescence-associated signature with upregulation of p21CIP1, p16INK4a, senescence-associated β-galactosidase, and the osteogenic marker RUNX2, along with decreased expression of Ki67, sirtuin 1, Nrf2, and MHY11. This imbalance reflects the accumulation of senescent cells and the development of an early vascular aging (EVA) phenotype [89].

Aging in CKD is amplified by sustained oxidative stress and chronic low-grade inflammation, commonly referred to as “inflammaging.” Senescent cell accumulation within the kidney further aggravates tissue injury by releasing pro-inflammatory mediators and proteolytic factors collectively known as the senescence-associated secretory phenotype (SASP) [90]. Telomere attrition, mitochondrial dysfunction, and impaired autophagy act in concert to promote cellular senescence in CKD, driving the accumulation of damaged proteins and organelles and thereby accelerating renal aging [91,92]. The pro-inflammatory secretome of senescent cells further aggravates renal fibrosis and systemic inflammation [93].

Beyond renal tissue, accelerated aging in CKD extends to other organ systems. Skeletal muscle loss (sarcopenia) is increasingly recognized as a hallmark of premature aging in these patients. Sarcopenia in CKD arises from a multifactorial interplay of uremic toxin accumulation, insulin resistance, chronic inflammation, and endocrine disturbances such as elevated cortisol, which together drive protein catabolism and impair muscle protein synthesis, as summarized in a recent narrative review [94]. In a 16-year longitudinal community-based cohort study, Lee et al. demonstrated that the coexistence of diabetes mellitus and CKD was synergically associated with accelerated muscle loss and increased risk of cachexia, underscoring the clinical consequences of this comorbidity on functional decline [95]. Since skeletal muscle mass is strongly associated with longevity and resilience against multimorbidity, sarcopenia in CKD represents not merely a clinical complication but also an independent predictor of all-cause mortality, as demonstrated in recent population-based data [53].

Neuroendocrine–immune dysregulation further accelerates aging in CKD. In a large cross-sectional study, higher metabolic scores for visceral fat were independently associated with CKD risk, supporting the link between adiposity, metabolic dysregulation, and loss of lean body mass [53]. Together with vascular senescence and immune dysfunction, these alterations contribute to a frailty-like state, further increasing vulnerability to adverse outcomes.

In summary, CKD exemplifies a systemic state of premature aging, driven by oxidative stress, inflammation, mitochondrial dysfunction, and neuroendocrine imbalance. Importantly, sarcopenia and loss of skeletal muscle integrity have emerged as key determinants of longevity, underscoring the need for therapeutic strategies that address not only renoprotection but also systemic aging hallmarks to improve survival and quality of life in CKD patients.

### 2.6. The Relationship Between Diet, Cortisol, and CKD

Diet exerts a significant influence on cortisol dynamics and HPA-axis activity, which is especially relevant in CKD. In the general population, chronic and acute stress have been shown to increase the intake of energy-dense “comfort foods,” partly due to their transient dampening effects on the stress-response network. Conversely, habitual dietary patterns rich in refined sugars and ultra-processed foods are associated with reduced cortisol reactivity, insulin resistance, systemic inflammation, and, ultimately, increased CKD risk [96,97]. In contrast, dietary interventions emphasizing whole foods, high polyphenol content, and Mediterranean-style eating show promise in reducing fasting morning cortisol and improving metabolic parameters, including kidney function [98,99].

In the CKD population, medical nutrition therapy—with careful management of protein, sodium, phosphorus, potassium, and calcium—can alleviate renal workload and contribute to overall metabolic balance [100]. A recent narrative review of individualized diets in CKD and kidney transplant patients reported that balanced Mediterranean and plant-based dietary patterns improved glycemic control, lipid profile, and blood pressure, which are indirect modulators of cortisol levels and systemic stress [101].

Moreover, plant protein–dominant diets and supplementation with ketoanalogues have been evaluated in CKD stages 3–4. In a randomized controlled trial, a vegetarian very low-protein diet supplemented with ketoanalogues slowed CKD progression and improved metabolic acidosis [102]. More recent reviews further emphasize that such individualized dietary strategies—including plant-based and low-protein patterns—can reduce dietary acid load and phosphorus burden, stabilize symptoms, and support overall metabolic health in CKD [103]. While direct evidence on cortisol modulation in these dietary trials is lacking, such metabolic benefits may indirectly mitigate chronic HPA axis overstimulation.

In summary, dietary patterns characterized by plant dominance, reduced ultra-processed food intake, and high antioxidant and fiber content represent a promising adjunctive strategy to attenuate HPA axis dysregulation in CKD. By reducing systemic inflammation, improving metabolic balance, and indirectly modulating cortisol exposure, these nutritional approaches may ultimately support renal function preservation and slow disease progression (Table 4).

## 3. Discussion

### 3.1. Integrated Mechanistic Perspective

This review highlights the interplay between oxidative stress, chronic low-grade inflammation, and dysregulation of the HPA/PNEI axis in the pathogenesis of CKD. While oxidative stress and inflammation have long been recognized as hallmarks of renal injury, accumulating evidence underscores the contribution of neuroendocrine dysregulation—particularly altered cortisol dynamics—in shaping the systemic environment that accelerates CKD progression. Together, these processes converge to amplify endothelial dysfunction, fibrosis, and metabolic imbalance. Importantly, this integrative view positions CKD not only as a renal disorder but as a systemic stress-related condition.

### 3.2. Biomarkers and Clinical Relevance

A wide range of biomarkers has been proposed to capture these interconnected mechanisms. Oxidative stress is reflected by MDA, AOPPs, and 8-OHdG; inflammation by IL-6, TNF-α, and IL-10; and stress physiology by serum, salivary, and hair cortisol. However, heterogeneity in study designs, small sample sizes, and methodological variability limit the comparability of findings. At present, no standardized biomarker panel is available for routine use. A combined approach that integrates markers of oxidative stress, inflammation, and cortisol may offer better predictive accuracy for CKD progression and comorbidities.

### 3.3. Therapeutic Implications

Several limitations in the available literature must be acknowledged. Most studies are cross-sectional or based on small patient cohorts, precluding causal inference. Cortisol measurement across different biological matrices lacks standardization, complicating the interpretation of results. Moreover, diet–cortisol interactions in CKD remain poorly characterized, and the role of gut microbiota in modulating systemic inflammation and stress responses is only beginning to be elucidated. These gaps emphasize the need for longitudinal and mechanistic studies to validate biomarkers and therapeutic strategies.

### 3.4. Future Directions

Future research should focus on the development of integrated biomarker panels that combine oxidative stress, inflammatory, and neuroendocrine markers for risk stratification. Standardized protocols for multimatrix cortisol assessment are needed to facilitate clinical translation. Randomized controlled trials testing mechanism-based dietary and pharmacological interventions will be crucial to establish causal links and optimize treatment strategies. Ultimately, adopting a multidimensional framework that incorporates stress physiology into nephrology could advance both personalized risk prediction and therapeutic approaches in CKD.

### 3.5. Limitations

This review has several limitations. First, most available studies are cross-sectional or small in scale, which restricts the strength of causal inference. Second, findings on oxidative and inflammatory biomarkers in CKD are sometimes heterogeneous, reflecting differences in patient populations, disease stages, and analytical methods. Third, evidence on long-term cortisol dynamics remains limited, and standardized measurement protocols are lacking. Finally, while this review aimed to integrate the most relevant literature published between 2010 and 2025, some emerging studies may not yet be available. Future large-scale longitudinal and interventional studies are needed to validate the role of cortisol, oxidative stress, and inflammatory markers in CKD progression and to clarify their clinical applicability.

## 4. Conclusions

Chronic kidney disease exemplifies a systemic disorder in which oxidative stress, chronic inflammation, and HPA/PNEI axis dysregulation act in concert to drive disease onset and progression. Cortisol, together with inflammatory and oxidative biomarkers, represents a promising avenue for the identification of high-risk patients and the development of multimarker panels for risk stratification. Beyond classical metabolic risk factors, lifestyle and dietary patterns exert important modulatory effects on stress physiology and may indirectly mitigate kidney injury.

Integrating biomarkers of oxidative stress, inflammation, and cortisol into clinical research and practice could enable earlier detection of maladaptive stress responses and guide more personalized interventions. Future work should prioritize longitudinal studies and mechanism-based therapeutic trials to clarify causal pathways and translate this integrative perspective into effective strategies for slowing CKD progression and improving patient outcomes.

## Figures and Tables

**Table 3:** Dysregulation of the HPA/PNEI axis in CKD: mechanisms and consequences.

Aspect	Mechanism/Effect	References
Cortisol secretion and circadian rhythm	Normally peaks in the morning and declines in the evening; in CKD the rhythm is blunted/phase-shifted with reduced morning peak and elevated evening levels.	[44]
Altered cortisol metabolism	Reduced renal clearance and increased 11β-HSD1 activity enhance local cortisol regeneration and impair HPA feedback.	[45]
Metabolic and cardiovascular effects	Elevated cortisol promotes visceral adiposity, insulin resistance, hypertension, and dyslipidemia—key risk factors for CKD.	[46,52,53,54]
Glucocorticoid resistance and inflammation	Chronic cortisol excess induces immune cell resistance, paradoxically enhancing IL-6, TNF-α, and CRP production.	[45,47]
PNEI crosstalk (SNS and RAAS)	Sympathetic overactivation increases RAAS signaling, blood pressure, and fibrosis; NADPH oxidases drive oxidative stress.	[9,48,49]
Immune imbalance	Cortisol and catecholamine excess alter T-cell differentiation and macrophage polarization, favoring Th1/Th17 and M1 phenotypes, sustaining inflammation.	[50,51]
Psychological/behavioral aspects	Dysregulated stress hormones linked with sleep disturbances and depression, further destabilizing HPA regulation.	[56,57]
Therapeutic implications	MR antagonists mitigate cortisol/aldosterone-driven injury; IL-6 inhibition and structured exercise improve vascular function, inflammation, and stress response.	[58,59,60,61,62,63]
Clinical relevance	Monitoring cortisol dynamics and HPA activity may allow early detection of maladaptive stress responses and guide personalized CKD interventions.	[9,44]

Legend: This table summarizes key mechanisms through which HPA/PNEI dysregulation contributes to CKD progression, integrating neuroendocrine, immune, metabolic, and psychosocial pathways.

**Table 4:** The relationship between diet, cortisol, and CKD: dietary patterns, mechanisms, and outcomes.

Dietary Pattern/Intervention	Effect on Cortisol/HPA Axis and CKD Outcomes	References
High intake of refined sugars and ultra-processed foods	Reduced cortisol reactivity, insulin resistance, systemic inflammation; ↑ CKD risk.	[96,97]
Mediterranean-style and polyphenol-rich diets	Lower fasting morning cortisol, improved metabolic parameters and kidney function.	[98,99]
Medical nutrition therapy (protein, sodium, phosphorus, potassium, calcium control)	Alleviates renal workload, improves metabolic balance in CKD.	[100]
Mediterranean and plant-based diets in CKD and transplant patients	Improve glycemic control, lipid profile, BP; indirectly modulate cortisol and systemic stress.	[101]
Vegetarian very low-protein diet + ketoanalogues (CKD stages 3–4)	Slows CKD progression, improves metabolic acidosis.	[102]
Plant-based/low-protein individualized diets	Reduce dietary acid load and phosphorus burden; stabilize symptoms, support metabolic health; indirect effects on HPA axis.	[103]

Legend: This table summarizes evidence on how different dietary patterns influence cortisol dynamics, systemic inflammation, and CKD outcomes. Plant-dominant, antioxidant-rich diets appear promising in attenuating HPA axis dysregulation and supporting renal protection. The arrow (↑) indicate an increase, such as elevated CKD risk or higher levels of the described parameter.

## Data Availability

No new data were created or analyzed in this study.

## Abbreviations

The following abbreviations are used in this manuscript:

Abbreviation	Full Term
8-OHdG	8-hydroxy-2′-deoxyguanosine
AhR	Aryl hydrocarbon receptor
AOPPs	Advanced oxidation protein products
BP	Blood pressure
CAT	Catalase
CKD	Chronic kidney disease
CRP	C-reactive protein
CVD	Cardiovascular disease
eGFR	Estimated glomerular filtration rate
eNOS	Endothelial nitric oxide synthase
GLP-1RA	Glucagon-like peptide-1 receptor agonist
GPx	Glutathione peroxidase
GSH	Reduced glutathione
HPA axis	Hypothalamic–pituitary–adrenal axis
HDL	High-density lipoprotein
IL	Interleukin (IL-1β, IL-6, IL-10)
LDL	Low-density lipoprotein
MAPK	Mitogen-activated protein kinase
MDA	Malondialdehyde
MHY11	Myosin heavy chain 11
m6A	N6-methyladenosine (RNA modification)
NADPH	Nicotinamide adenine dinucleotide phosphate
NF-κB	Nuclear factor kappa-light-chain-enhancer of activated B cells
NLRP3	NOD-like receptor family pyrin domain-containing 3 inflammasome
NOX	NADPH oxidase (isoforms: NOX4, NOX5)
Nrf2	Nuclear factor erythroid 2–related factor 2
OBS	Oxidative balance score
PCOS	Polycystic ovary syndrome
PNEI	Psycho-neuro-endocrine-immune axis
PPARs	Peroxisome proliferator-activated receptors
RAAS	Renin–angiotensin–aldosterone system
ROS	Reactive oxygen species
RUNX2	Runt-related transcription factor 2
SASP	Senescence-associated secretory phenotype
SGLT2(i)	Sodium–glucose cotransporter-2 (inhibitors)
SII	Systemic immune-inflammation index
SIRT1	Sirtuin 1
SOD	Superoxide dismutase
SREBP-1	Sterol regulatory element-binding protein 1
TBARS	Thiobarbituric acid reactive substances
TGF-β	Transforming growth factor beta
Th1/Th17	T helper 1/T helper 17 cells
TLR(s)	Toll-like receptor(s)
TNF-α	Tumor necrosis factor alpha
